# Synthesis of Acylated Xylan-Based Magnetic Fe_3_O_4_ Hydrogels and Their Application for H_2_O_2_ Detection

**DOI:** 10.3390/ma9080690

**Published:** 2016-08-11

**Authors:** Qing-Qing Dai, Jun-Li Ren, Feng Peng, Xiao-Feng Chen, Cun-Dian Gao, Run-Cang Sun

**Affiliations:** 1 State Key Laboratory of Pulp and Paper Engineering, South China University of Technology, Guangzhou 510640, China; fedaiqing@mail.scut.edu.cn (Q.-Q.D.); c.xiaofeng01@mail.scut.edu.cn (X.-F.C.); gaocundian@gmail.com (C.-D.G.); 2 Beijing Key Laboratory of Lignocellulosic Chemistry, Beijing Forestry University, Beijing 100083, China; fengpeng@bjfu.edu.cn (F.P.); rcsun3@bjfu.edu.cn (R.-C.S.)

**Keywords:** xylan, acylated xylan, magnetic hydrogels, Fe_3_O_4_ nanoparticles, H_2_O_2_ detection

## Abstract

Acylated xylan-based magnetic Fe_3_O_4_ nanocomposite hydrogels (ACX-MNP-gels) were prepared by fabricating Fe_3_O_4_ nanoctahedra in situ within a hydrogel matrix which was synthesized by the copolymerization of acylated xylan (ACX) with acrylamide and *N*-isopropylacrylamide under ultraviolet irradiation. The size of the Fe_3_O_4_ fabricated within the hydrogel matrix could be adjusted through controlling the crosslinking concentrations (C). The magnetic hydrogels showed desirable magnetic and mechanical properties, which were confirmed by XRD, Raman spectroscopy, physical property measurement system, SEM, TGA, and compression test. Moreover, the catalytic performance of the magnetic hydrogels was explored. The magnetic hydrogels (C = 7.5 wt %) presented excellent catalytic activity and provided a sensitive response to H_2_O_2_ detection even at a concentration level of 5 × 10^−6^ mol·L^−1^. This approach to preparing magnetic hydrogels loaded with Fe_3_O_4_ nanoparticles endows xylan-based hydrogels with new promising applications in biotechnology and environmental chemistry.

## 1. Introduction

Hydrogels possessing three-dimensional networks can absorb enormous amounts of biological fluids or water but still keep their structural integrity [1], which provides a liquid-like microenvironment where small molecules can easily diffuse and be transported. In recent years, multifunctional nanocomposite smart hydrogels have received great attention because of their outstanding properties which provide them with the novel features of the nanomaterials [2], such as photo-thermal, magnetic, and catalytic properties, as well as multiple environmental response behaviors (the reversible change of volume in response to external stimuli such as temperature or salt) [3,4,5,6].

Compared with other kinds of nanocomposite hydrogels, notable achievements have been made in the development of magnetic hydrogels because of their enormous potential in biomedical and technological applications, such as cancer therapy, wastewater treatment, and drug release [7,8,9]. It was reported that magnetite nanoparticles could be widely used to oxidize organic substrates in the treatment of wastewater, or used as detection tools instead of natural peroxidases, because of their intrinsic enzyme mimetic activity [10]. Compared with natural enzymes such as horseradish peroxidase (HRP), Fe_3_O_4_ nanoparticles are well suited to be used as catalysts for H_2_O_2_ oxidation due to their properties of robustness, repeatability, low cost, easy recovery, and a wide range of applications in environmental and chemical biotechnology [11]. For example, during wastewater treatment, the reduced toxicity of organic substrates could be realized and color change occurred after the catalytic oxidation of H_2_O_2_ [10]. In biotechnology, H_2_O_2_ catalysis can be employed for the trace detection of H_2_O_2_ released from cells or produced by glucose oxidation, which is important for human health and disease [12,13].

Xylan, as a major component of hemicellulose, is the rich, inexhaustible, and renewable hetero-polysaccharide consisting of various different sugar units with short-branched chains, yet it is poorly utilized as green raw material, which is the promising natural polymer behind cellulose [14,15]. Xylan can be chemically modified to form functional polymers by different approaches, such as etherification, esterification, oxidation, polymer blending, grafting copolymerization, and compounding with other functional components to increase its reactivity [16,17,18,19,20,21,22]. Recently, more and more attention has been focused on the synthesis of hemicellulose or hemicellulose derivative based hydrogels because it could reduce the corresponding cost, improve the biocompatibility, and broaden the application of hydrogels in various biomedical and industrial fields [23,24,25,26]. For example, Gao et al. [25] synthesized a pH/temperature dual response xylan-based hydrogel. Cao et al. [27] designed a novel xylan-based hydrogel which showed good photo-responsive properties by free radical copolymerization. Zhao et al. [28] prepared a type of electrically conductive hemicellulose hydrogel (ECHH) with macroporous structures by crosslinking *O*-acetyl-galactoglucomannan (AcGGM) with epichlorohydrin in the presence of conductive aniline pentamer (AP), through a facile and mild method in water at room temperature. To improve the reactivity of hemicellulose, many studies have introduced alkenyl groups onto the backbone structure which could promote the reactions with other monomers to obtain the biocompatible macromolecular copolymers. Peng et al. [29] designed a new kind of hemicellulosic derivative containing polymerizable carbon–carbon double bonds (C=C) via introduction of methacryloyl groups onto xylan-type hemicellulose. Multi-responsive hydrogels were designed by copolymerizing glycidyl methacrylate (GMA)-modified xylan-type hemicellulose with 4-[(4-acryloyloxyphenyl)azo] benzoic acid (AOPAB), and these hydrogels showed sensitive responses to pH, light, and water/ethanol alternating solutions, which were indicative of broad prospects in the application of light-controlled systems [27]. However, few comparative studies have been conducted on the preparation and characterization of acylated xylan-based hydrogels with magnetic-response behavior as well as their catalytic activity toward the detection of H_2_O_2_.

In this study, modified xylan based hydrogels possessing magnetic properties were designed by fabricating Fe_3_O_4_ nanoctahedra in situ within a hydrogel matrix which was synthesized by the copolymerization of acryloyl chloride modified xylan (ACX) with acrylamide and *N*-isopropylacrylamide using *N*,*N′*-methylenebis-acrylamide (MBA) as a cross-linker under ultraviolet (UV) irradiation. Furthermore, their application for H_2_O_2_ detection was also explored. The mechanical properties of acryloyl chloride modified xylan based magnetic hydrogels (ACX-MNP-gels) were discussed. The magnetic hydrogels were characterized by X-ray diffraction (XRD), Raman spectroscopy, Scanning electron microscopy (SEM), Physical property measurement system (PPMS) and Thermogravimetric analysis (TGA). In addition, a comparative experiment was conducted to study the influence of different crosslinking concentrations (C) on the properties of the hydrogels. The as-prepared magnetic hydrogels were used to catalyze the oxidation of a peroxidase substrate (3,3′,5,5′-tetramethylbenzidine, TMB) (10 mL) by H_2_O_2_ to yield the oxidized colored product which provided a colorimetric detection of H_2_O_2_.

## 2. Experimental Section

### 2.1. Materials

Beech wood xylan (*M_w_* of 130,000 g·mol^−1^) was purchased from Sigma Aldrich (Karlsruhe, Germany) and used as received. *N*-isopropylacrylamide (NIPAm, 98%), *N*,*N′*-methylenebisacrylamide (MBA, 98%), acrylamide (AM, 98%), and dimethylaminopyridine (DMAP, 99%) were supplied by Aladdin Reagent Company Limited (Shanghai, China). Acetylsalicylic acid (99%), dimethyl formamide (DMF, 99%), and LiCl were purchased from Macklin Reagent Company Limited (Shanghai, China). Dimethoxybenzoin (DMPA, 99%), *N*-methyl-2-pyrrolidone (NMP, 99%), FeSO_4_·7H_2_O, FeCl_3_·6H_2_O, NH_3_·H_2_O, H_2_O_2_, and ethanol were purchased from Guangzhou Chemical Reagent Factory (Guangzhou, China). 3,3′,5,5′-tetramethylbenzidine (TMB) was obtained from Yuanye Bio-engineering Material Company (Shanghai, China).

### 2.2. Preparation of ACX and Xylan or ACX Based Magnetic Gels

#### 2.2.1. Preparation of ACX

0.66 g xylan was dissolved in 10 mL distilled water [30], and then 20 mL DMF was added under a 10 min continuous magnetic stirring. After xylan was dissolved completely, water in the solution was removed by the rotary evaporation apparatus. Finally, 0.1 g LiCl, 0.033 g DMAP, 15 mL DMF and 2.437 mL acryloyl chloride (1:3 molar ratio of hydroxyls on xylan to acryloyl chloride) were added to the mixture under stirring and the final solution was kept at 70 °C for 40 min [31,32]. The mixture was precipitated with 100 mL 95% (w/w) ethanol after the reaction, and then separated by centrifugation and further washed with 75% (w/w) ethanol three times to remove unreacted reagents. Finally, the modified xylan (ACX) was obtained after oven drying. In addition, the degree of substitution (DS) of ACX was 0.63, which was determined by the contents of C, H, N, and S of the sample conducted via the elemental analysis [33]. The samples were ground into powder and dehydrated at 50 °C for 24 h. The DS values were calculated as follows [29]:

(1) $$\mathrm{DS}=\frac{C\%\times 132-60}{36-55\times C\%}$$

where *C*% is the carbon percentage of ACX tested by elemental analysis. 132 and 55 are the molecular weights (g/mol) of the xylose unit in xylan and the acryloyl group, respectively. 60 and 36 are the total molecular weights (g/mol) of the carbon element in the xylose unit and acryloyl group, respectively.

#### 2.2.2. Preparation of Xylan or ACX Based Magnetic Hydrogels

**Scheme 1:**
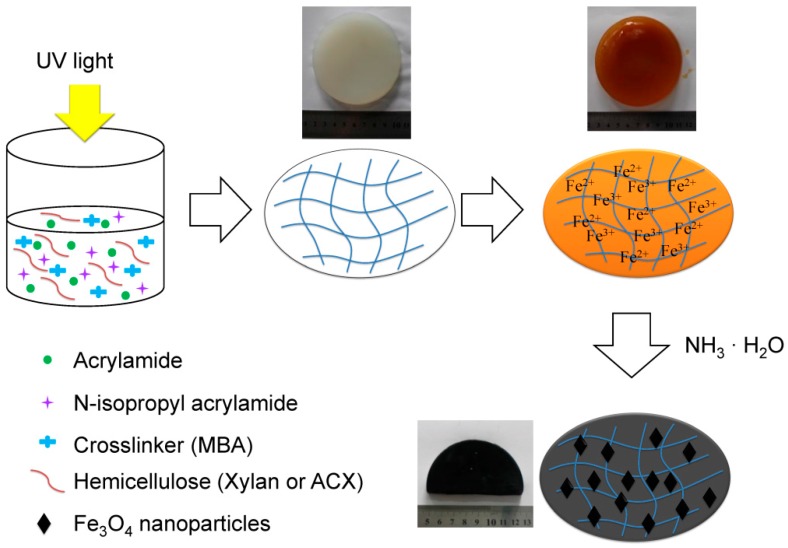
Schematic process for the preparation of magnetic Fe_3_O_4_ nanocomposite hydrogels.

Xylan based hydrogels (Xylan-gels) or ACX based hydrogels (ACX-gels) containing no Fe_3_O_4_ particles were prepared according to the method reported by Gao et al. [25]. An aqueous solution containing 5 wt % xylan (or ACX) was heated at 85 °C for 1.5 h under continuous stirring and subsequently cooled down to 50 °C. NIPAm, AM, and MBA (crosslinking concentrations were 2.5, 5.0, 7.5, 10.0 wt %, respectively, based on the xylan or ACX weight) were added into the mixture as a crosslinking agent. After bubbling N_2_ for 20 min, DMPA (5.0 wt %, based on the xylan or ACX weight) was first dissolved in the NMP solution (2.5 wt %), and then added into the mixture as the photosensitizer. When the solution had achieved a homogeneous state, the mixture was added into a quartz mould under ultraviolet irradiation (365 nm, 40 W) at room temperature for 6 h. Subsequently, the samples were well-sealed at room temperature for 8 h to ensure further polymerization and to complete crosslinking of the networks. The detailed synthesis conditions are shown in Table 1. After the end of the reaction, the transparent hydrogels with different C (C = 2.5 wt %, 5.0 wt %, 7.5 wt % and 10.0 wt %) were cut into cubes and immediately immersed in an aqueous solution containing iron ions with a molar ratio of (Fe^3+^)/(Fe^2+^) = 2:1 for 12 h. The COO^−^ groups on the chain of xylan could promote the uptake and enrichment of the iron ions inside the magnetosome vesicles [34,35]. The hydrogel disks loaded with iron ions were subsequently transferred into a NH_3_·H_2_O aqueous solution and kept at a room temperature for 1 h under a nitrogen atmosphere, resulting in the precipitation of Fe_3_O_4_ nanoparticles (MNPs) within the hydrogel matrix. The preparation process of the magnetic hydrogels (Xylan-MNP-gels or ACX-MNP-gels) is schematically depicted in Scheme 1.

### 2.3. Fourier Transform Infrared Spectroscopy (FTIR) Analysis

Fourier transform infrared spectroscopy (FTIR) of xylan or ACX was performed by means of a Fourier transform spectrophotometer (Nicolet 750, Sarasota, FL, USA), with a resolution of 4 cm^−1^, taking over 32 scans per sample, ranging from 400 to 4000 cm^−1^. All samples were dehydrated in an oven at 50 °C before testing, and then the finely ground samples were mixed with KBr to be pressed into a plate for the measurement.

### 2.4. Morphology of Hydrogels

The morphology of the hydrogels was examined by scanning electron microscopy (SEM, Hitachi S3700, Tokyo, Japan) with an accelerating voltage of 10 kV. The magnetic hydrogels were dehydrated by vacuum freeze-drying before testing.

### 2.5. X-ray Diffraction (XRD) Analysis

X-ray diffraction (XRD) patterns were collected on an X-ray diffractometer (Bruker, model D8 advance, Karlsruhe, Germany) with Cu K(*α*) radiation (*λ* = 1.54060 Å) at an accelerating voltage of 40 kV and a current of 40 mA. The spectra were collected from 2*θ* in the range of 20°–80°.

### 2.6. Raman Spectra

Raman spectra (H.J.Y, LabRAM Aramis, Paris, France) were excited by 632.8 nm radiation from an argon ion laser with an output power of 20 mW, and a typical acquisition time of 10 s.

### 2.7. Magnetic Measurements

The dried magnetic hydrogels were ground into powder and then measurements were carried out with a physical property measurement system (PPMS-9, Quantum Design Inc., San Diego, CA, USA) at room temperature.

### 2.8. Determination of Swelling Degree of Hydrogels

Equilibrium swelling experiments were conducted by the gravimetric method in water with desired pH at different temperatures to study the swelling capacity of the prepared hydrogels. All samples were dehydrated and then immersed in water to achieve an equilibrium swelling state in the temperature range from 25 to 37 °C. Subsequently, the absorbed moisture on the surface of swollen samples was removed using a filter paper to ensure an accurate measurement. The determination of all samples was conducted by three parallel measurements.

Generally, the swelling ratio (SR) and the equilibrium swelling ratio (S_eq_) were defined as follows [36]:

(2) $$\mathrm{SR}=\frac{W_{t}-W_{o}}{W_{O}}$$

(3) $$S_{\mathrm{eq}}=\frac{W_{eq}-W_{o}}{W_{O}}$$

where *W*_t_ is the weight of the swollen hydrogels, *W*_o_ is the weight of the dried hydrogels, and *W*_eq_ is the equilibrium weight of the swollen hydrogels.

### 2.9. Thermogravimetric Analysis

A simultaneous thermal analyzer (TGA Q500, TA Instruments, New Castle, DE, USA) under a nitrogen flow of 20 mL·min^−1^ was utilized to discern the thermogravimetric properties of the hydrogels. 10 mg dried hydrogel samples were ground into powder and heated from 50 to 600 °C at a 10 °C·min^−1^ heating rate in an open alumina crucible.

### 2.10. Catalytic Experiment and H_2_O_2_ Detection

The catalytic activity of the magnetic hydrogels with different crosslinking concentrations (C) were explored using TMB as the substrate in the presence of H_2_O_2_ [5], and were incubated in a 40 °C water bath for 1 h. TMB was chosen due to the high affinity of Fe_3_O_4_ nanoparticles for TMB. Catalytic experiments were conducted using the magnetic hydrogel pieces loaded with Fe_3_O_4_ nanooctahedra immersed in the substrate solution with various H_2_O_2_ concentrations (5 uM–200 mM). The magnetic hydrogel disks were removed using a magnet after the reaction was completed. The substrate solutions were analyzed using a UV/Vis spectrophotometer (SHIMADZU UV1800, Kyoto, Japan) with a wavelength-scan mode of 650 nm.

## 3. Results and Discussion

### 3.1. FTIR Analysis

**Figure 1:**
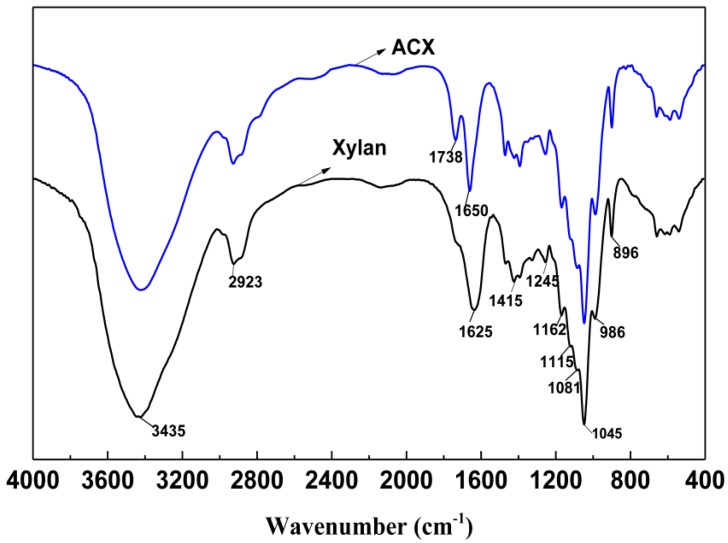
FTIR spectra of xylan and ACX.

The FTIR spectra of xylan and ACX are illustrated in Figure 1. The absorption bands at 3435, 2923, 1625, 1415, 1245, 1162, 1115, 1081, 1045, 986, and 896 cm^−1^ are attributed to xylan [25]. The broad absorption peak at 3431 cm^−1^ is assigned to the stretching vibrations of –OH groups of xylan. The decreased intensity of ACX at 3435 cm^−1^ revealed the decrease of hydroxyl groups of xylan, which confirmed that the chemical modification occurred on the hydroxyl groups of xylan. The band at 2923 cm^−1^ is assigned to the C–H stretching vibration of alkane in xylan. C–O–C and C–O stretching with some contribution of the OH bending mode are contributable to the band at 1162 cm^−1^. The prominent band at 1045 cm^−1^ belongs to the C–O–C stretching of pyranoid ring xylan. A sharp band at 896 cm^−1^ originates from the *β*-glucosidic linkages between xylose units. In contrast, the intense signals at 1738 cm^−1^ and 1650 cm^−1^ in the spectrum of ACX are ascribed to the stretching vibrations of C=C and C=O (in ester group) originating from acryloyl chloride [27]. These results confirmed that acryloyl groups were introduced to the xylan chains successfully.

### 3.2. The Crystal Structure Analysis of Fe_3_O_4_

**Figure 2:**
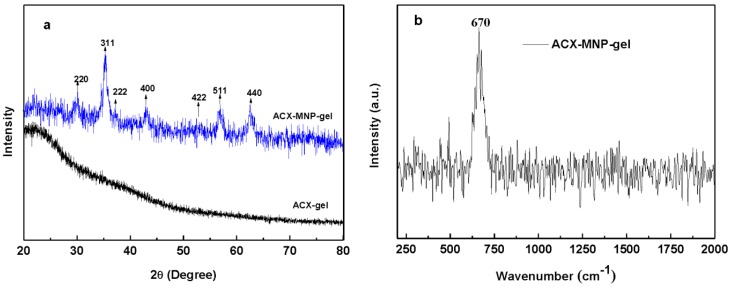
The crystal structure of Fe_3_O_4_ nanoparticles in situ fabricated within the ACX-MNP-gel matrix (C = 5.0 wt %). (**a**) XRD spectra; (**b**) Raman spectra.

In order to investigate the phase and structure of the prepared hydrogels, dried magnetic samples were firstly ground into powder and then used for XRD measurements. There were no peaks in the spectrum of a typical ACX-gel, indicating an amorphous structure. However, for the spectrum of ACX-MNP-gels (Figure 2a), the broad peaks at 2*θ* = 30.25°, 35.70°, 43.16°, 53.94°, 57.42°, and 62.83° were observed which are attributed to the (220), (311), (400), (422), (511), and (440) planes of Fe_3_O_4_ respectively [37,38]. All of the diffraction peaks in Figure 2a of the ACX-MNP-gels can be identified as inverse spinel Fe_3_O_4_ with a face-centered cubic structure. However, the XRD pattern of *γ*-Fe_2_O_3_ is similar to Fe_3_O_4_. Therefore, Raman spectroscopy, which is used to effectively investigate the structure of iron oxides, was utilized to further study the crystal structure of Fe_3_O_4_. The Raman spectrum shows a single and intense peak at 670 cm^−1^ (Figure 2b), which is consistent with the value of Fe_3_O_4_ reported in the literature [39], but is different from that of *γ*-Fe_2_O_3_ which has three prominent peaks around 700, 500, and 350 cm^−1^ [40]. Therefore, the nanocrystals in the hydrogels were definitely determined to be Fe_3_O_4_ rather than *γ*-Fe_2_O_3_. Moreover, the crystal sizes of the ACX-MNP-gels were determined by the Debye-Scherrer equation (Equation (4)) from the XRD data [41]. Table 2 clearly reflects that there was a continuous increase in particle size when C increased from 2.5 wt % to 7.5 wt %. However, when C reached 10.0 wt %, the crystal size began to decline, which could be explained by a higher crosslinking density that would result in an inhibition of the growth of Fe_3_O_4_ particles. The Debye-Scherrer equation is as follows,

(4) D=Kλ/(βcosθ)

where D is the average crystal diameter, *K* is a constant referring to the shape of the crystallites (*K* = 0.94), *λ* is the wavelength of X-rays employed (*λ* = 0.154056), *β* is the corrected peak width (full width at half-maximum), and *θ* is the diffraction angle (2*θ* = 35.70°).

### 3.3. Morphological Analysis

**Figure 3:**
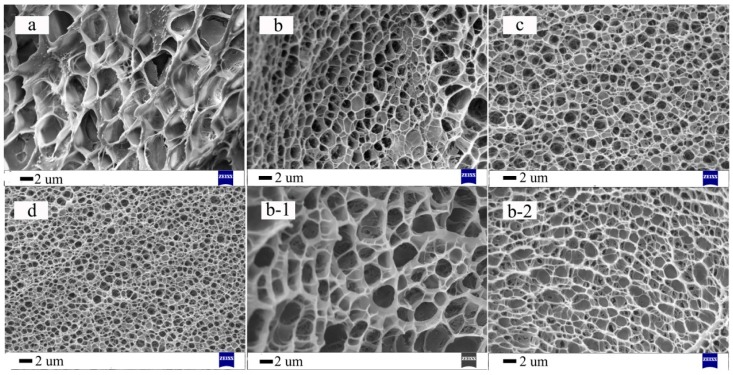
The surface morphology of hydrogels. (**a**) ACX-gel with C = 2.5 wt %; (**b**) ACX-gel with C = 5.0 wt %; (**c**) ACX-gel with C = 7.5 wt %; (**d**) xylan-gel with C = 10.0 wt %; (**b-1**) xylan-gel with C = 5.0 wt %; (**b-2**) AXC-MNP-gel with C = 5.0 wt %. Scale bar: 2 µm.

The SEM images demonstrate the surface morphology of the hydrogels. It is obvious that all the hydrogels presented micron-size porous structures similar to a honeycomb. The SEM images clearly show a higher intensive aperture with the increase of C (Figure 3a–d). This phenomenon indicates that a higher C could contribute to an intensive structure and smaller aperture [42]. However, with the same C, ACX-gels (Figure 3b) displayed denser and more homogeneous porous networks in comparison with xylan-gels (Figure 3b-1) possessing a relatively macroporous structure, suggesting ACX-gels had a higher crosslinking density. The SEM image in Figure 3b-2 shows that the apertures of the hydrogel were clogged compared with Figure 3b, indicating that Fe_3_O_4_ particles were successfully loaded into the hydrogels.

**Figure 4:**
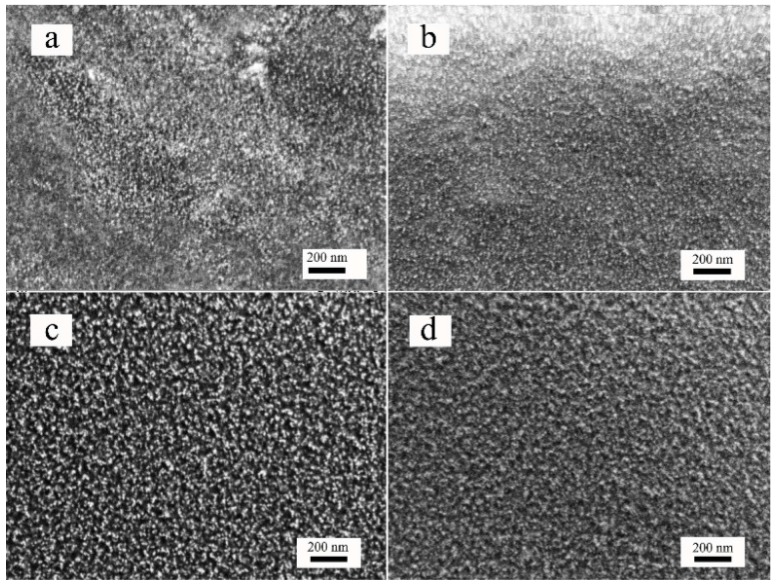
SEM images of Fe_3_O_4_ nanoparticles contained within ACX-MNP-gels. (**a**) C = 2.5 wt %; (**b**) C = 5.0 wt %; (**c**) C = 7.5 wt %; (**d**) C = 10.0 wt %; Scale bar: 200 nm.

Figure 4 shows the SEM images of Fe_3_O_4_ nanoparticles loaded within magnetic hydrogels with different C. Obviously, the size of the Fe_3_O_4_ nanoparticles could be adjusted by controlling the crosslinking concentration of the hydrogel. ACX-MNP-gels with C = 7.5 wt % provided the most suitable microenvironment for anisotropic growth, in line with the TGA results (Fe_3_O_4_ content) and the XRD data in Table 2. With a low crosslinking concentration, irregular shaped nanoparticles with a smaller size could be obtained [43,44]. It was reported that anisotropic growth begins to appear with the increase of C, resulting in an octahedral geometry. However, when C continued to increase to a certain value, the anisotropic growth disappeared and the nanoparticles gained a sphere-like morphology [5]. As a result, it was distinctly revealed that the crosslinking concentration of the hydrogel plays an important role in manipulating the Fe_3_O_4_ nanoparticles.

### 3.4. Swelling Behavior

**Figure 5:**
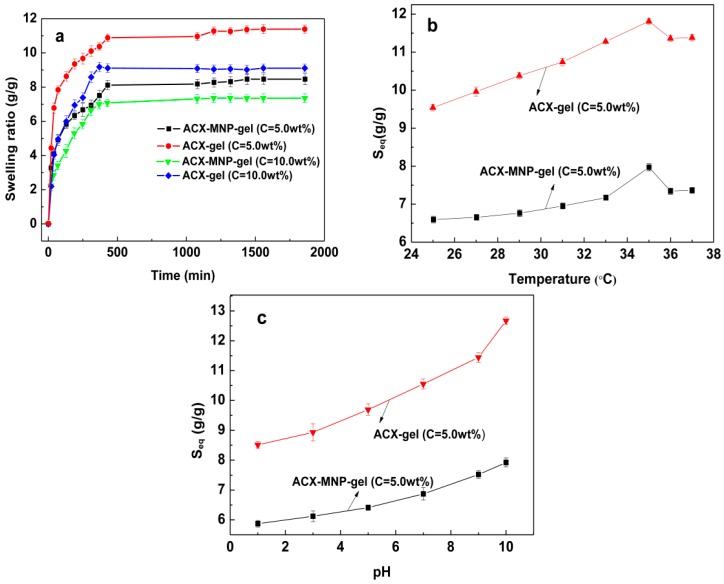
(**a**) The swelling ratio of ACX-gels and ACX-MNP-gels with different C as a function of time; (**b**) The S_eq_ of ACX-gels and ACX-MNP-gels as a function of temperature in neutral pH; (**c**) The S_eq_ of ACX-gels and ACX-MNP-gels as a function of pH at 30 °C.

The swelling ratio (SR) is an important property used to evaluate the swelling capacity of hydrogels. The as-prepared ACX-gels and ACX-MNP-gels with different C as a function of time, temperature, and pH are shown in Figure 5. In Figure 5a, the SR of all hydrogels increased quickly within the first 2 h and then the swelling speed slowed down and reached an equilibrium state. The equilibrium swelling ratios (S_eq_) of ACX-gels and ACX-MNP-gels were 11.38 and 8.46 when C was 5.0 wt %. When C increased to 10.0 wt %, the S_eq_ of ACX-gels and ACX-MNP-gels were 9.08 and 7.35, respectively. In comparsion, the ACX-gels could achieve a higher S_eq_. In addition, the data indicated that gels with a lower C could reach a higher S_eq_ at a faster swelling speed. Moreover, the S_eq_ decreased with an increase of C, because a higher C contributes to a higher intensive aperture, which could inhibit the permeation of water [45]. In addition, it is speculated that the presence of Fe_3_O_4_ particles was unfavorable for the stretching of polymer chain segment motion.

The influence of temperature on the S_eq_ of ACX-gels and ACX-MNP-gels is illustrated in Figure 5b. The S_eq_ of all samples showed a similar trend, that first increased and then decreased with a further increase in temperature. The maximum values were achieved at 35 °C. This phenomenon showed that the lower critical solution temperature (LCST) of these hydrogels occurred at around 35 °C and hydrogels shrunk sharply after 35 °C which resulted in a lower S_eq_ [46,47]. For ACX-gels, the S_eq_ increased steadily from 9.54 to 11.81 before 35 °C but had a rapid deswelling response at 36 °C. Upon further increase of the temperature to 37 °C, the S_eq_ increased slightly. The S_eq_ of the ACX-MNP-gels increased slowly up to 33 °C, and then swelled up with an increase from 33 to 35 °C proportionately. There was no obvious change from 36 to 37 °C. Furthermore, the maximum S_eq_ of the ACX-gels was 11.36, which was higher than that of the ACX-MNP-gels. This is confirmed by Figure 3b-2, that the hydrogel matrix was clogged with Fe_3_O_4_ nanoparticles which led to a decrease of the S_eq_.

Figure 5c shows the S_eq_ of ACX-gels and ACX-MNP-gels immersed in the solution with different pH at 30 °C. The S_eq_ of all samples increased along with the increase of pH from 1 to 10, which is due to the changes in the intermolecular forces and the swelling osmotic pressure [25]. The acidic environment provided a favorable condition for the formation of hydrogen bonds in the hydrogel matrix and the solution system which could inhibit the swelling behaviors of hydrogels. However, when the pH achieved an alkaline condition, the ionization of the –OH groups on ACX promoted space in the networks due to the electrostatic repulsion in alkaline conditions [48].

### 3.5. Magnetic Properties of Hydrogels

**Figure 6:**
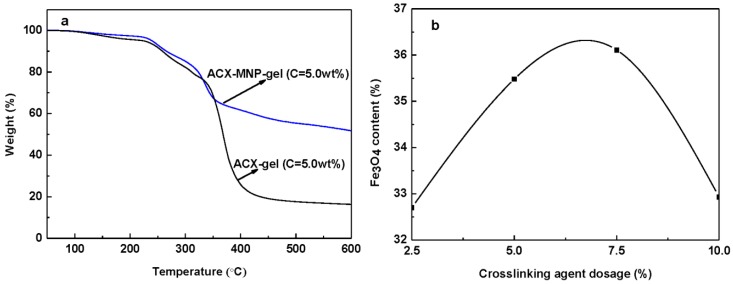
(**a**) Thermogravimetric curves of dehydrated hydrogels with C = 5.0 wt %; (**b**) The content curve of Fe_3_O_4_ in ACX-MNP-gels with different crosslinking concentrations.

Thermogravimetric analysis shown in Figure 6a was used to determine the thermal stability of the hydrogels and their loading capacity of Fe_3_O_4_ nanoparticles. The freeze-dried hydrogels were dehydrated below 100 °C to achieve a complete evaporation of moisture. The weight loss stage occurred at around 230 °C and 300 °C, as a consequence of the dehydroxylation and decarboxylation of ACX and organic polymer [49]. The weight-loss process that occurred at 300–400 °C was due to the cleavage of the C–C backbone in hydrogels. In addition, the degradation rate of ACX-MNP-gels was higher than that of ACX-gels when the temperature was 300–350 °C, due to the destruction of its network under the alkaline environment when loading the Fe_3_O_4_ particles, which led to a lower thermal stability. When the temperature rose above 400 °C, the weight of the sample related to Fe_3_O_4_ gradually decreased and leveled off. It was reported that the slight weight-loss at the last stage was due to the decomposition of polymer produced reducing gas, which may react with Fe_3_O_4_ [50]. The residue of ACX-gels was mainly carbon black ash. Therefore, the difference in level off values could be used to determine the loading capacity of Fe_3_O_4_ nanoparticles [50]. The loading capacity of Fe_3_O_4_ nanoparticles of various magnetic hydrogels are shown in Figure 6b. First, the loading capacity of Fe_3_O_4_ increased along with the increase in crosslinking concentrations. However, the loading capacity of Fe_3_O_4_ decreased when C surpassed 7.5 wt %. This could be explained by the fact that at a higher C, which indicates a more compact network structure of hydrogels, there was more surface area that promoted the growth of Fe_3_O_4_ particles [43,51]. When C increased to 10.0 wt %, the compact structure led to the lower swelling ability and the lower loading capacity of Fe_3_O_4_ particles. It was speculated that the loading capacity of Fe_3_O_4_ particles was affected by the surface area and swelling ratio, and when C reached 7.5 wt %, the maximum loading capacity of Fe_3_O_4_ particles could be obtained. In Figure 4c, Fe_3_O_4_ nanoparticles were distributed evenly in the networks of hydrogels, along with a slight aggregation due to the existence of ACX. It is speculated that the hydrogen bonding interaction in or between molecules contributed to the aggregation of Fe_3_O_4_ nanoparticles.

**Figure 7:**
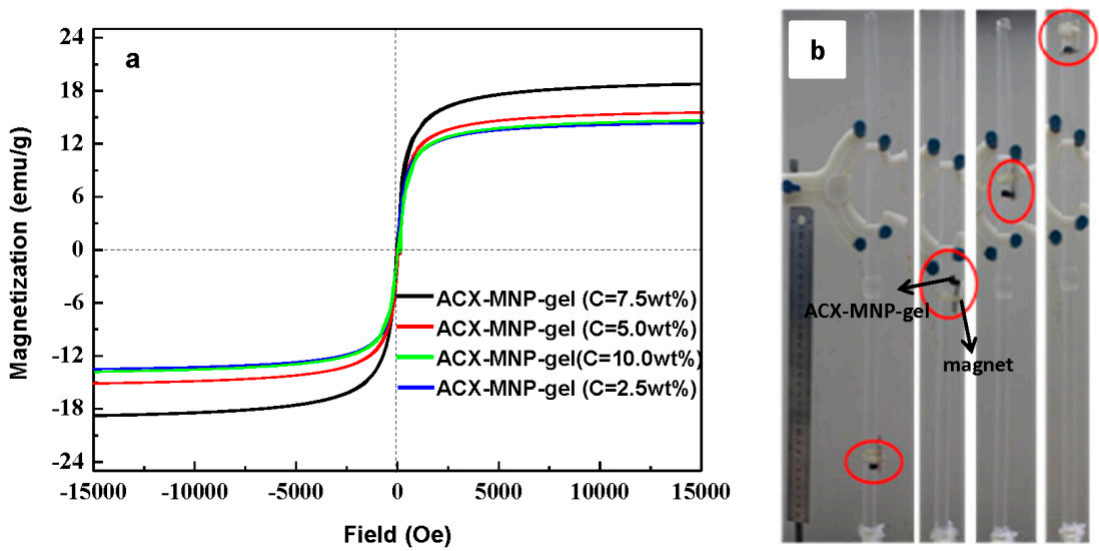
(**a**) The magnetization curve of the dehydrated ACX-MNP-gels loaded with Fe_3_O_4_ nanooctahedra; (**b**) Photographs of the ACX-MNP-gels in a quartz tube full of water with a magnet on the outside of the quartz tube.

The hysteresis loop of the magnetic hydrogels detected by VSM analysis is shown in Figure 7a. It shows ferromagnetic properties of the hydrogels with saturation magnetization of 14.09, 15.02, 18.74, and 13.61 emu/g with C from 2.5 wt %, 5.0 wt %, 7.5 wt %, and 10.0 wt %, respectively. There was no hysteresis phenomenon, and the magnetic hydrogels presented with low coercivity and remanence, and exhibited typical superparamagnetism. These results proved that the saturation magnetization was affected by multiple factors. Fe_3_O_4_ particles were grown in the networks of hydrogels with various C, which indicated that the crosslinking concentration could affect the magnetic properties of Fe_3_O_4_ by influencing the size or morphology of the Fe_3_O_4_ particles. In addition, both the saturation magnetization and the size of Fe_3_O_4_ particles first increased and then reduced with the increase of C. This was consistent with previous reports that the saturation magnetization increases with an increase in size [52,53]. The presence of polymers within the hydrogels contributed to a reduction of the saturation magnetization. Therefore, the magnetic composites exhibited a much lower saturation magnetization compared with the pure Fe_3_O_4_ nanoparticles. Figure 7b shows that the magnetic hydrogel piece could be removed easily in the quartz tube full of water by an external magnetic field, implying that it could be easily recovered and has a potential application in environmental chemistry.

### 3.6. Mechanical Property Analysis

**Figure 8:**
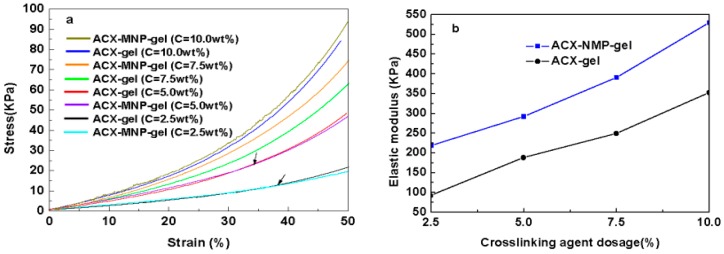

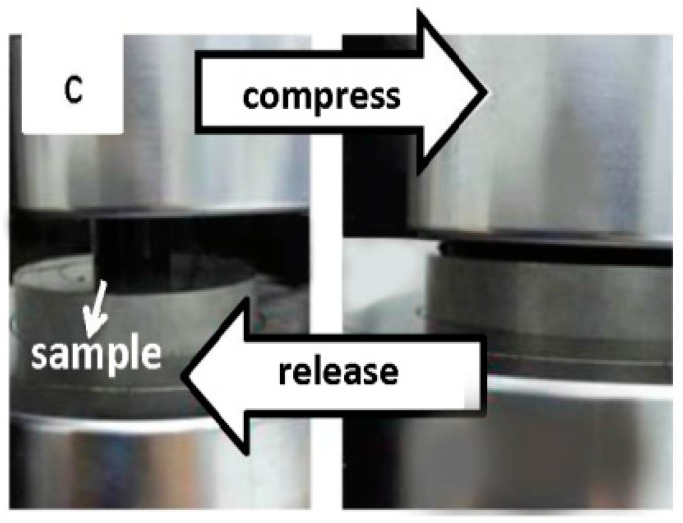
(**a**) Compressive stress-strain curves of ACX-gels and ACX-MNP-gels; (**b**) the elastic modulus of ACX-gels and ACX-MNP-gels with different C; (**c**) the ACX-MNP-gels were highly compressed and quickly recovered to their original state.

Figure 8 shows the compressive properties of hydrogels to evaluate the density of the hydrogel networks, and it shows an almost linear type growth. Compared with our previous work [25], the strength of the ACX-based hydrogels was evident. When the crosslinking concentration was below 5.0 wt %, the compression strength of the ACX-MNP-gels were superior to the ACX-gels in the beginning, but when the compressive strain was above 30%, the ACX-gels exhibited a higher compression strength. One possible explanation was that part of the network structure of the hydrogels was broken down in the alkaline environment when the Fe_3_O_4_ particles were fabricated within the hydrogel network via co-precipitation, resulting in a decrease of the compression strength. With an increase of C, the hydrogel network would become more compact. It is obvious in Figure 8b that the elastic modulus obtained from the linear region of ACX-MNP-gels could achieve double the elastic modulus of the ACX-gels at the same C, indicating that the participation of Fe_3_O_4_ particles could enhance the mechanical properties of hydrogels. From Figure 8c, the ACX-MNP-gels were highly compressed and quickly recovered to their original state.

### 3.7. Catalytic Experiments and H_2_O_2_ Detection

**Figure 9:**
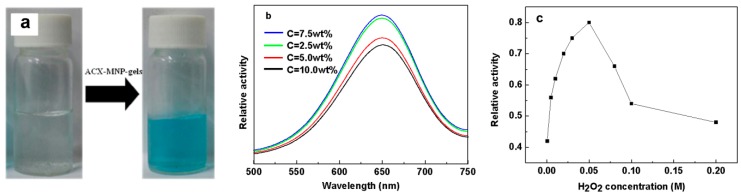
(**a**) Color variations in the catalytic experiment; (**b**) Relative activity of the ACX-MNP-gels with different C; (**c**) H_2_O_2_ concentration-response curve using the ACX-MNP-gels (C = 7.5 wt %) as a catalyst under the same reaction conditions and with a H_2_O_2_ concentration range of 5 uM to 200 mM.

Catalytic experiments were conducted with various magnetic nanocomposite hydrogels, and the content of Fe_3_O_4_ within the magnetic hydrogels was kept consistent prior to the catalytic activity characterization. The as-prepared magnetic nanocomposite hydrogels were then used to catalyze the oxidation of a peroxidase substrate TMB (10 mL) by H_2_O_2_ (Equation 5) which provides a colorimetric detection of H_2_O_2_. Fe_3_O_4_ nanoparticles showed a high affinity for TMB [10]. The colorless solution containing TMB and H_2_O_2_ displayed a negligible absorption in the range of 500 nm to 750 nm. By contrast, in the presence of the magnetic nanocomposite hydrogels, the solution turned a blue color (Figure 9a) and exhibited a similar characteristic maximum absorbance at around 650 nm (Figure 9b). This indicates that the intrinsic peroxidase-like properties of the Fe_3_O_4_ nanoparticles could still be reserved after in situ preparation within the hydrogel. Moreover, the magnetic hydrogels could be easily removed from the solution with a magnet at the end of the reaction.

(5) $$H_{2}O_{2}+\mathrm{TMB}\;\overset{{\mathrm{Fe}}_{3}O_{4}}{\to }\;H_{2}\text{O}+\mathrm{oxidized}\;\mathrm{TMB}$$

The magnetic hydrogels with different C exhibited different absorbance intensities (Figure 9b). It is considered that both the size and morphology of the Fe_3_O_4_ fabricated within the hydrogels had great influences on the catalytic performance of the magnetic hydrogels [5]. Smaller sized Fe_3_O_4_ has a larger surface-to-volume ratio that could promote its catalytic activity. Moreover, diverse morphologies lead to the exposure of the crystal planes with different energies which could influence the catalytic activity. The magnetic hydrogel with C = 7.5 wt % showed the highest catalytic activity, implying that C = 7.5 wt % provided the most suitable microenvironment for anisotropic growth. In addition, the magnetic hydrogel with C = 2.5 wt % displayed the second highest catalytic activity, and the magnetic hydrogel with C = 5.0 wt % presented the third highest catalytic activity, which was slightly higher than that of the magnetic hydrogel with C = 10.0 wt %. Their different catalytic performances could be ascribed to the distinct morphology of the Fe_3_O_4_ nanocomposites according to the previous report [5]. One possible explanation for these findings was that the magnetic hydrogel with C = 5.0 wt % had an octahedral morphology and the magnetic hydrogel with C = 10.0 wt % had a sphere-like morphology. In addition, the magnetic hydrogel with C = 2.5 wt % possessed a smaller average size, resulting in a relatively high catalytic activity. The H_2_O_2_ concentration-response curve is shown in Figure 9c. The ACX-MNP-gels showed a sensitive catalytic response towards the H_2_O_2_ detection even at a concentration of 5 × 10^−6^ mol·L^−1^. When the concentration of H_2_O_2_ was lower than 50 mM, the absorbance intensity increased with the increase of H_2_O_2_ concentration. The absorbance intensity decreased above a H_2_O_2_ concentration of 50 mM due to the repulsion which caused white sediment. This result suggests that the magnetic hydrogel could be used as a detection tool in the fields of biotechnology and environmental chemistry.

## 4. Conclusions

In conclusion, ACX-based hydrogels possessing magnetic properties were synthesized successfully by grafting copolymerization under ultraviolet irradiation. Results demonstrated that introducing functional groups on xylan could contribute to higher intensive aperture networks and to more intense mechanical properties of the ACX-MNP-gels. Moreover, the size of the Fe_3_O_4_ nanoparticles could be controlled by adjusting the crosslinking concentration of the hydrogels. The magnetic hydrogels presented intrinsic peroxidase-like activities. With a higher catalytic activity, the magnetic xylan-based hydrogel had a sensitive response to H_2_O_2_ detection even at a low concentration of 5 × 10^−6^ mol·L^−1^. In addition, the magnetic nanocomposite hydrogels could be recovered more effectively and could be easily used as a carrier. Finally, the magnetic hydrogels possessed good magnetic/temperature dual-response, which could have a potential application in the biotechnology and environmental chemistry fields.

## Figures and Tables

**Table 1:** Synthesis conditions of xylan-gels and ACX-gels.

Xylan-Gels		ACX-Gels	
NIPAm/xylan (g/g)	0.1	NIPAm/ACX (g/g)	0.1
AM/xylan (g/g)	5.0	AM/ACX (g/g)	5.0
MBA/xylan (g/g)	2.5–10.0	MBA/ACX (g/g)	2.5–10.0
Initiator (%)	2.5	Initiator (%)	2.5

**Table 2:** The average crystallite size of ACX-MNP-gels from the XRD data.

Samples	C	Crystallite Size (nm)
a	2.5 wt %	7.20
b	5.0 wt %	9.32
c	7.5 wt %	10.24
d	10.0 wt %	9.63
